# Solid-State Heating Synthesis of Poly (3,4-Ethylenedioxythiophene)/Gold/Graphene Composite and Its Application for Amperometric Determination of Nitrite and Iodate

**DOI:** 10.1186/s11671-017-2338-8

**Published:** 2017-10-17

**Authors:** Ahmat Ali, Yu Zhang, Ruxangul Jamal, Tursun Abdiryim

**Affiliations:** 1 Key Laboratory of Petroleum and Gas Fine Chemicals, Educational Ministry of China, College of Chemistry and Chemical Engineering, Xinjiang University, Urumqi, 830046 People’s Republic of China; 2 Key Laboratory of Functional Polymers, Xinjiang University, Urumqi, 830046 People’s Republic of China

**Keywords:** Solid-state method, Poly (3,4-ethylenedioxythiophene), Composite, Amperometry, Ion detection

## Abstract

A ternary composite of poly (3,4-ethylenedioxythiophene)/gold/graphene (PEDOT/Au/GO) for promising electrochemical sensor was synthesized by solid-state heating method. The interaction between the PEDOT, Au, and GO explored for detection of nitrite and iodate. It was found that the PEDOT/Au/GO composite had shale-like morphology with a uniform distribution of gold nanoparticles. Electrochemical experiments showed that the PEDOT/Au/GO composite modified electrode exhibited good electrocatalytic activity toward determination of iodate. The amperometric experiments at the PEDOT/Au/GO/GCE revealed that a good linear relationship existed between peak current and the concentration in the range of 100–1000 μM with the detection of 0.53 and 0.62 μM (S/N = 3) for nitrite and iodate, respectively. Moreover, the current response of PEDOT/Au/GO/GCE for nitrite and iodate at 10 μM was up to 9.59 and 11.47 μA, respectively.

**Figure:**
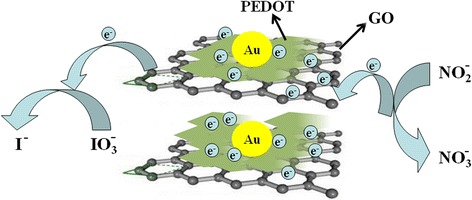
Mechanisms of the direct electron transfer between ion(nitrite or iodate)and the PEDOT/Au/GO composite

Mechanisms of the direct electron transfer between ion(nitrite or iodate)and the PEDOT/Au/GO composite

## Background

Nitrite (NO_2_^−^) is ubiquitous within environmental, food, and agricultural products, which was recognized to exist in physiological systems when ingest compounds contain NO_2_^−^ [1, 2]. NO_2_^−^ can react with amines to form carcinogenic nitrosamines, and the continuous ingestion of these ions can be harmful to animal and human health [3–5]. Also with the other ion press close to our daily life, iodate (IO_3_^−^), the iodized salt, is recognized as the most successful strategy for the prevention of iodide deficiency disorders. However, an excess of IO_3_^−^ can produce goiter and hypothyroidism as well as hyperthyroidism [6, 7]. Therefore, many techniques have been developed for NO_2_^−^ and IO_3_^−^ detection [8], including spectroscopic [9], chromatographic [10], chemiluminescence [11], electrochemical [12–15], and capillary electrophoresis methods [16]. Among them, the electrochemical method has been widely used due to its high sensitivity, simplicity, rapidness, and low cost. Generally, the electrodes have modified with nanostructured metal (such as Pt, Au), metal oxide (such as WO_3_, RuO_2_), and carbon nanomaterials, and which have been extensively investigated for the development of effective electrochemical sensors [17–20]. Among them, Au nanoparticles have broad applications in the field of electrochemical sensors with its ideal catalytic activity, sensitivity, biocompatibility, interface-dominated properties, excellent conductivity, and high signal to noise ratio. However, high cost, poor selectivity, and instability of Au make it unsuitable for practical applications [21].

Recently, the conducting polymer/gold hybrid materials have been extensively investigated to obtain new kind composite materials with synergetic or complementary behaviors [22, 23]. As one of the typical and important part of conducting polymers, poly (3,4-ethylenedioxythiophene) (PEDOT) has wide applications in the field of displays, smart windows, sensors, capacitors, batteries, and photovoltaic devices [24–26]. Generally, in chemically synthesized PEDOT/Au composites, electrocatalytic performances of the composite could be enhanced through Au–S(thiophene) interactions and the activation of metal ion coordination [27, 28]. And many reports have been published for the preparation of binary PEDOT/Au composites [29, 30].

In recent years, most of the researches focus on the preparation of graphene/conducting polymer-based ternary composites because of graphene-based carbon materials have high surface area, unique electronic transport property, high electrocatalytic activity, and good chemical stability [31, 32]. These unique characteristics of graphene-based carbon materials possibly bring unique chemical structures and more superior performance to the composites [33].

Yao et al. synthesized a PANI/MWNTs/Au composite sensor for detection of NO_2_^−^, and the current response was about 2.8 μA for 10 μM NO_2_^−^ [34]. Xue et al. prepared a ternary nanocomposite of gold nanoparticles/polypyrrole/graphene by facile wet-chemical routes and found that as-prepared composites have a good electrocatalytic activity toward the glucose with its high sensitivity [35]. In this case, research on the preparation, structure, and properties of graphene-based ternary nanocomposites will be very interesting and challenging in the fields of sensors. However, the conventional chemical and electrochemical technique for ternary nanocomposite is usually complicated and tedious. Therefore, cost-effective, clear, green, simple, and high-efficiency synthetic methods are desirable.

Herein, we report the fabrication of a ternary composite (PEDOT/Au/GO) of poly (3,4-ethylenedioxythiophene), gold nanoparticles, and graphene for promising electrochemical sensor by solid-state heating method. For comparison, the pure PEDOT and binary composite (PEDOT/Au) were also synthesized in the similar manner. The PEDOT/Au/GO and PEDOT/Au composite have been used for the electrochemical sensitive determination of iodate. And the PEDOT/Au/GO composite was selected for evaluating its potential application as electrochemical sensor for detection of nitrite and iodate on the basis of systematic studies on the amperometric determination of nitrite and iodate.

## Experimental

### Chemicals and Reagents

3,4-Ethylenedioxythiophene (EDOT) was obtained from Shanghai Aladdin Reagent Company (China), and it was purified by distillation under reduced pressure and stored in a refrigerator prior to use. Chloroauric acid hydrated (HAuCl_4_·4H_2_O) was purchased from Shanghai Aladdin Reagent Company (China). Graphene (GO) was purchased from Strem Chemicals Inc. (USA). All other reagents were of analytical grade and used as supplied without further purification. 2,5-Dibromo-3,4-ethylenedioxythiophene was synthesized according to the previous report [36].

### Synthesis of the PEDOT/Au/GO and PEDOT/Au composites

Before the synthesis of composites, the Au nanoparticle sol solution was prepared in advance. The Au nanoparticle sol solution was prepared by reducing HAuCl_4_ with NaBH_4_ as reductant. A typical preparation of Au nanoparticle sol solution was as follows: 60 mg of HAuCl_4_·3H_2_O was added to 100 mL of water to create HAuCl_4_ solution. A total of 3.4 mL of aqueous solution of Na_3_C_6_H_5_O_7_ (1%) was then added to the 40 mL of HAuCl_4_ solution under vigorous stirring for 10 min. The 1.2 mg NaBH_4_ was then quickly added, and the color of the solution immediately turned into a purple.

**Fig. 1:**
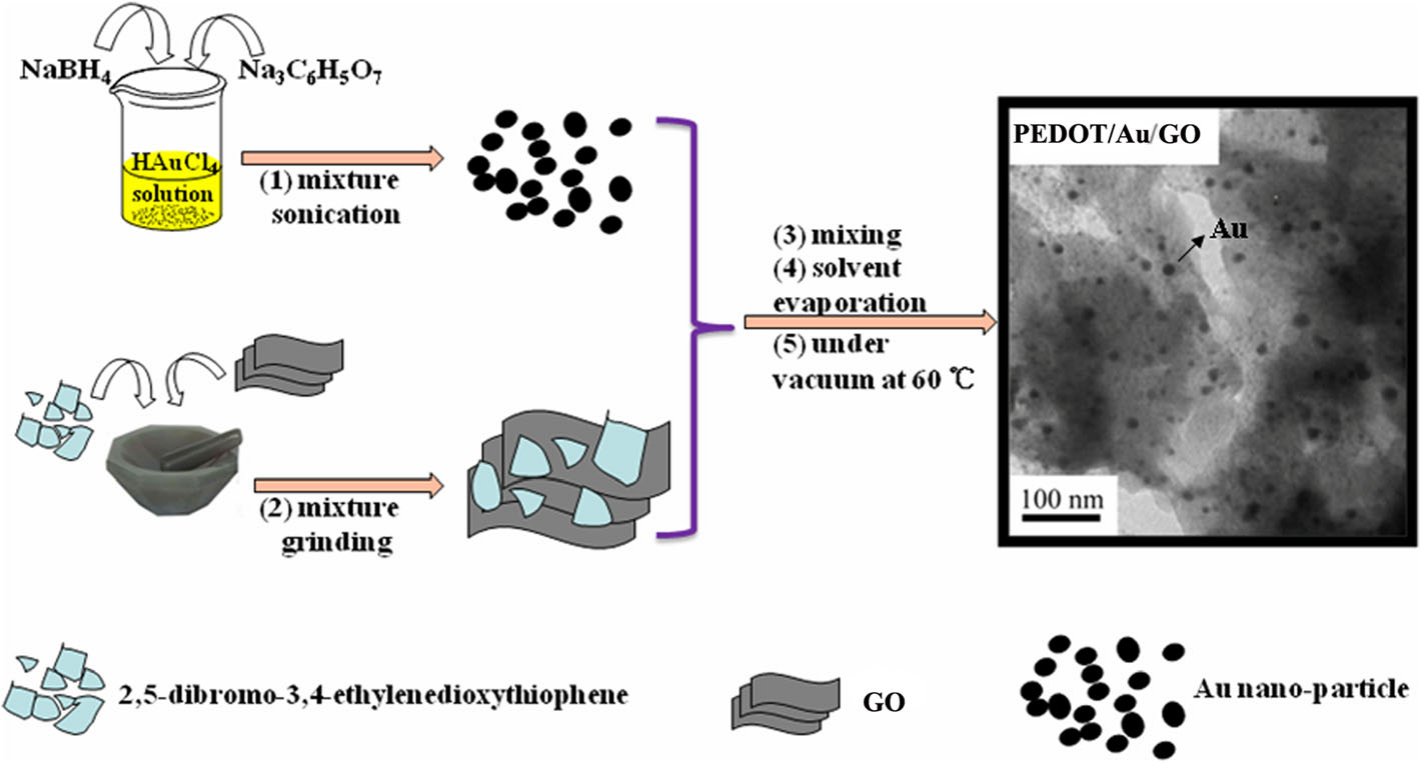
Schematic representation of the formation process of the PEDOT/Au/GO

A typical solid-state heating synthesis of PEDOT/Au/GO composite was as follows (Fig. 1): a mixture of 0.5 g (2 mmol) monomer (2,5-dibromo-3,4-thylene dioxythiophene) and 10 mg GO in 30 mL chloroform were ultrasonicated for 30 min to facilitate monomer to adsorb on the surface of GO. The mixture was then allowed to evaporate the chloroform. The residue was put in a mortar followed by constant grinding for 5 min. Then the mixture was added to the Au nanoparticle sol solution and stirred for 10 min. The mixture was then filtered and washed by distilled water, at last kept in a vacuum oven at 60 °C for 24 h. The obtained product was denoted as PEDOT/Au/GO composite.

For comparison, the binary composite (PEDOT/Au) and pure PEDOT were also synthesized in the similar manner.

### Structure Characterization

Fourier-transform infrared (FTIR) spectra of the samples were recorded on a BRUKER-QEUINOX-55 FTIR spectrometer using KBr pellets. UV-vis spectra of the samples were recorded on a UV-visible spectrophotometer (UV4802, Unico, USA). The samples for TEM measurements were prepared by placing a few drops of products ethanol suspension on copper supports and performed on a Hitachi 2600 electron microscope. The elemental content of the sample was characterized by energy-dispersive X-ray spectroscopy (EDS), which was taken on a Leo1430VP microscope with operating voltage 5 kV. EDX experiments were carried out with a pellet which was pressed at 200 MPa and then adhered to copper platens.

### Measurement of Electrocatalytic Activity

Cyclic voltammetry (CV) and amperometric *i*–*t* curve were performed on electrochemical workstation CHI 660C (ChenHua Instruments Co., Shanghai, China). Three-electrode system was employed to study the electrochemical performance of composite. Pt electrode was used as a counter electrode and saturated calomel electrode (SCE) as a reference electrode. PEDOT/Au/GO composite modified GCE (glassy carbon electrode; diameter = 3 mm) was used as a working electrode. The working electrode was fabricated by placing 5 μL of 30 mg/L PEDOT/Au/GO composite suspension (The PEDOT/Au/GO composite was dispersed in water to create suspension (30 mg/L).) on a bare GCE surface and air dried for 10 min. All the experiments were carried out at ambient temperature and air atmosphere.

## Results and Discussion

**Fig. 2:**
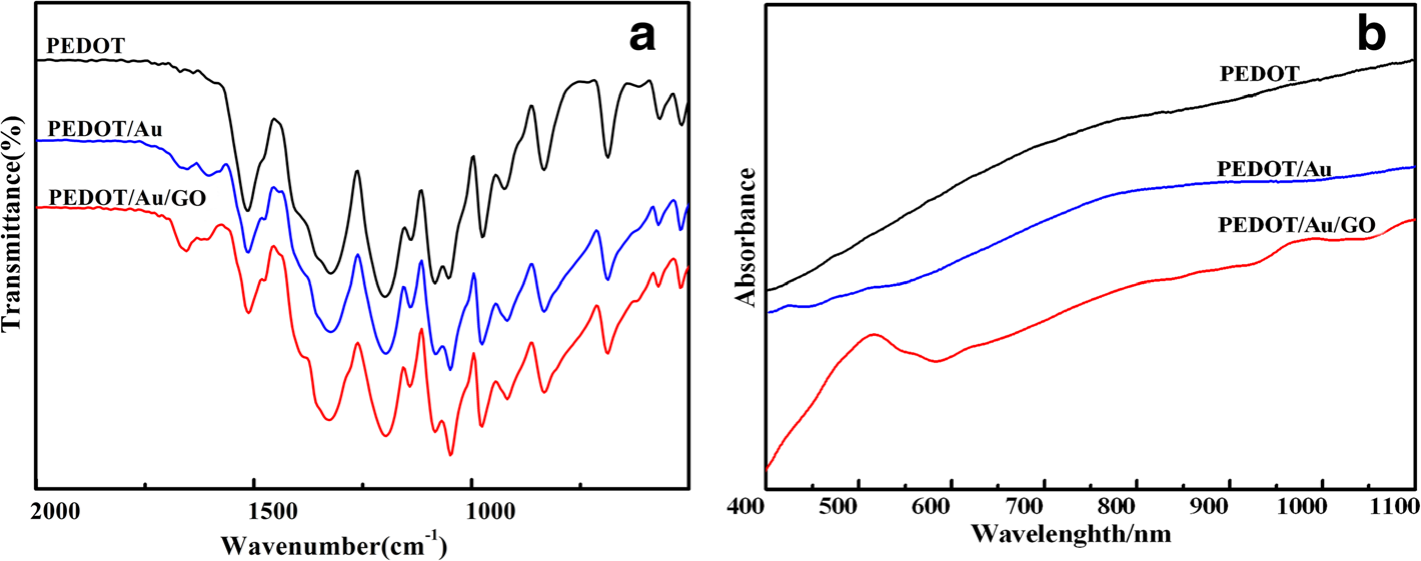
FTIR (**a**) and UV-vis (**b**) spectra of PEDOT, PEDOT/Au, and PEDOT/Au/GO

Figure 2a represents the FTIR spectra of PEDOT, PEDOT/Au, and PEDOT/Au/GO. As can be seen in Fig. 2a, the spectrum of PEDOT/Au/GO and PEDOT/Au composites are similar to that of pure PEDOT, indicating a successful formation of polymer in composite. The two bands appearing at ~ 1514 and ~ 1324 cm^−1^ are assigned to the asymmetric stretching mode of C=C and inter-ring stretching mode of C–C, respectively. The bands appearing at ~ 1198, ~ 1140, and ~ 1084 cm^−1^ are attributed to the C–O–C bending vibration in ethylenedioxy. These results are in good agreement with the previous reported FTIR spectra of PEDOT [37]. Although the spectra of PEDOT/Au/GO and PEDOT/Au composites are similar to that of pure PEDOT, several discrepancies occur between pure PEDOT and composites. According to the previous report, the polymerization degree of polythiophene can be evaluated from the ratio of integration of the infrared bands at 690 and 830 cm^−1^ [38, 39], and the higher degree of polymerization can be resulted from relatively lower value of that intensity ratio. Therefore, it can be deduced from Fig. 2a that the polymerization degree of the PEDOT/Au/GO, PEDOT/Au, and PEDOT is in the order of PEDOT/Au/GO > PEDOT/Au > PEDOT, which suggests that the PEDOT/Au/GO has a higher polymerization degree than PEDOT/Au and PEDOT. Furthermore, this result indicates that the presence of GO in reaction medium can play positive role in increasing the polymerization degree of PEDOT in composite matrix.

Figure 2b shows the UV-vis absorption spectra of PEDOT, PEDOT/Au, and PEDOT/Au/GO. As shown in Fig. 2b, the PEDOT displays broad absorption peak starting at ~ 500 nm and extending into the near-infrared region. This absorption feature, known as a “free carrier tail,” correlates with the conductivity of the polymers. The presence of this absorption peak has been shown to correspond to the polymer having a longer conjugation length and greater order, which allows for greater mobility of charge carriers [40, 41]. In the case of composites, the PEDOT/Au exhibits a similar absorption feature to that of PEDOT, while PEDOT/Au/GO displays a absorption peak (π-π* transition) at ~ 500 nm along with a free-carrier tail extending into the near-infrared region [37, 40, 42]. This phenomenon further implies that there is strong interaction between aromatic regions of the non-covalent graphene and quinoid rings of PEDOT [43, 44].

**Fig. 3:**
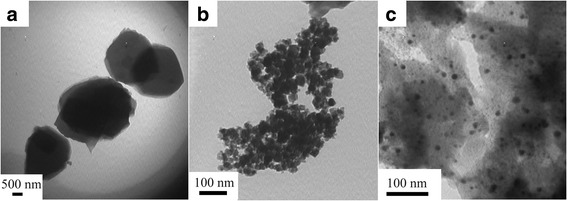
TEM images of **a** PEDOT, **b** PEDOT/Au, and **c** PEDOT/Au/GO

Figure 3 shows the transmission electron micrograph (TEM) images of PEDOT, PEDOT/Au, and PEDOT/Au/GO. As depicted in Fig. 3a, b, pure PEDOT exhibits shale-like morphology with layered structure, while the PEDOT/Au composite had granular-like morphology mixing from PEDOT and Au nanoparticles with an average size of 50 nm. However, in the case of PEDOT/Au/GO composite (Fig. 3c), it is found that the composite had shale-like morphology with a uniform distribution of gold nanoparticles (dark-shaded nanoparticles). Furthermore, the shale-like morphology of PEDOT/Au/GO composite is constructed from light-shaded and dark-shaded layered structure, which can be attributed to the GO and PEDOT, respectively. These results imply that the GO and Au nanoparticles are not simply mixed up or blended with the PEDOT, suggesting that the GO and Au nanoparticles (average size of 10~15 nm) are embedded in composite matrix. This uniform distribution of GO and Au nanoparticles in composite may be related to the shale-like morphology of PEDOT, which can bring some possibility for formation of lamellar structures from incorporation of PEDOT and GO, and leads a large surface area for uniform distribution Au nanoparticles.

**Fig. 4:**
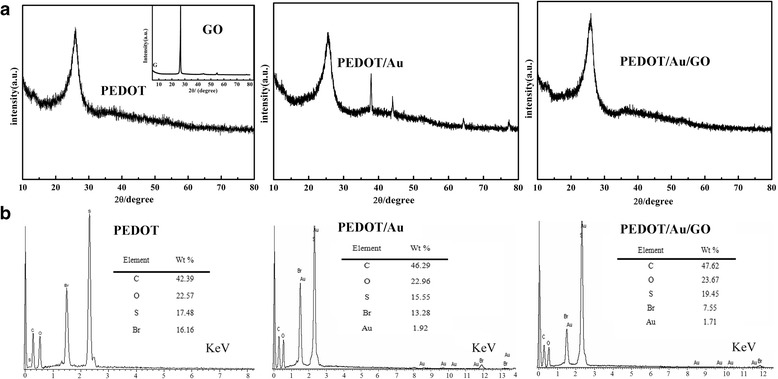
XRD (**a**) and EDX (**b**) of PEDOT, PEDOT/Au, and PEDOT/Au/GO

Figure 4a indicates the XRD patterns of PEDOT, PEDOT/Au, and PEDOT/Au/ GO. In addition, to study the element percentage of Au, energy-dispersive X-ray (EDX) spectroscopies of PEDOT, PEDOT/Au, and PEDOT/Au/GO are also shown in Fig. 4b. As depicted in Fig. 4a, the PEDOT, PEDOT/Au, and PEDOT/Au/GO display broad diffraction peaks with low intensity at 2*θ*~25.9°, which can be associated to the intermolecular spacing of polymer backbone or assigned to the (020) reflection [45]. In addition, the composite shows sharp diffraction peak at 2*θ*~26°, indicating the existence of GO in composite [46]. In the case of PEDOT/Au/GO composite, the characteristic diffraction peak of PEDOT (2*θ*~25.9°) is overlapped with that of GO (2*θ*~26.6°). The XRD pattern of composite indicates that the presence of characteristic diffraction peaks of Au (four peaks with low intensity at 2*θ* values of 37.9° and 43.7°), which correspond to Bragg’s reflections from the (111) and (200) planes of Au [47], suggesting the successful incorporation of Au in the composite, which is in accordance with the result of EDX (Fig. 4b) of PEDOT/Au (presence of 1.92 wt% Au). However, there is no obvious diffraction peak for Au in PEDOT/Au/GO, which is not matched with the result of EDX (Fig. 4b) of PEDOT/Au/GO (presence of 1.71 wt% Au). This may be attributed to the small particle size and high dispersion of Au nanoparticles in PEDOT/Au/GO composite, and this phenomenon is similar to the observation in Au/Zn nanocomposite, which did not show any diffraction peak for Au nanoparticles [47].

**Fig. 5:**
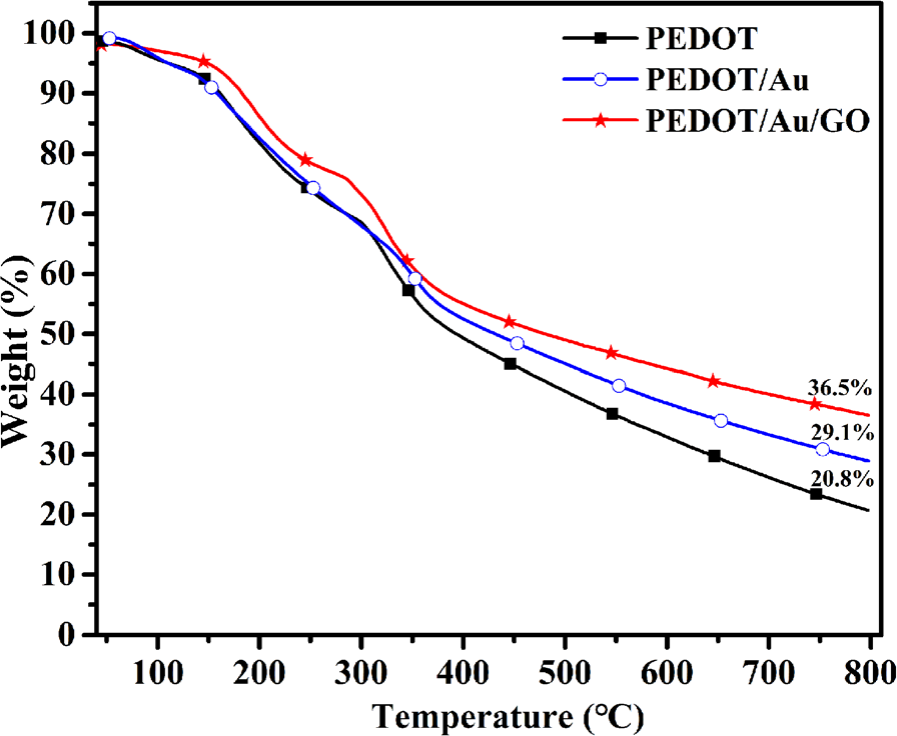
TGA curves of PEDOT, PEDOT/Au, and PEDOT/Au/GO

Thermogravimetric analysis of PEDOT, PEDOT/Au, and PEDOT/Au/GO are shown in Fig. 5. It is clear that these samples undergo three-step weight loss behaviors. The first step weight loss at 40–104 °C is due to the loss of trace of trapped water or moisture from the polymer chain. The second step weight losses occur at 112 to 323 °C with the weight loss of 24.78% (PEDOT), 24.33% (PEDOT/Au), and 19.17% (PEDOT/Au/GO), respectively. This is due to the loss of low molecular weight polymer. In the third step, polymer undergoes degradation after 323 °C. This result signpost that polymer is stable up to 323 °C. And present residual weight percentages of 20.8% (PEDOT), 29.1% (PEDOT/Au), and 36.5% (PEDOT/Au/GO) after 800 °C. These results suggest that presence of the Au and GO can enhance the thermo stabilities of the composites.

**Fig. 6:**
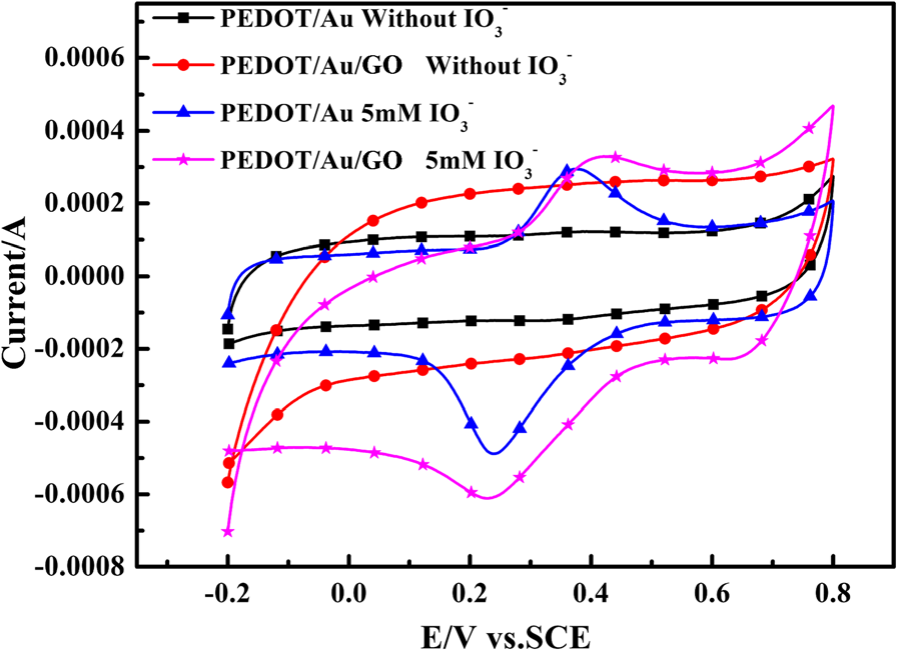
Cyclic voltammograms of PEDOT/Au/GO/GCE and PEDOT/Au/GCE in 0.1 M H2SO4 solution containing 5 mM iodate

To evaluate the potential application of PEDOT/Au/GO and PEDOT/Au composites as electrochemical sensor, the iodate (IO_3_^−^) is selected as testing specie for electrochemical experiment. Figure 6 shows cyclic voltammograms of PEDOT/Au/GO and PEDOT/Au composites in 0.1 M H_2_SO_4_ solution containing 5 mM iodate. As shown in Fig. 6, there is no oxidation/reduction peak in the both case of PEDOT/Au/GO (PEDOT/Au/GO/GCE) and PEDOT/Au modified glass carbon electrode (PEDOT/Au/GCE) without adding IO_3_^−^. When the IO_3_^−^ is added, both composites display a couple of oxidation/reduction peaks, and the reduction peak current value is higher than that of respective oxidation peak, which is resulted from the reduction of IO_3_^−^ to I^−^ [48]. Furthermore, the highest reduction current intensity occurs in the case of PEDOT/Au/GO/GCE, suggesting that PEDOT/Au/GO/GCE has an enhanced electrochemical catalytic activity than PEDOT/Au/GO.

**Fig. 7:**
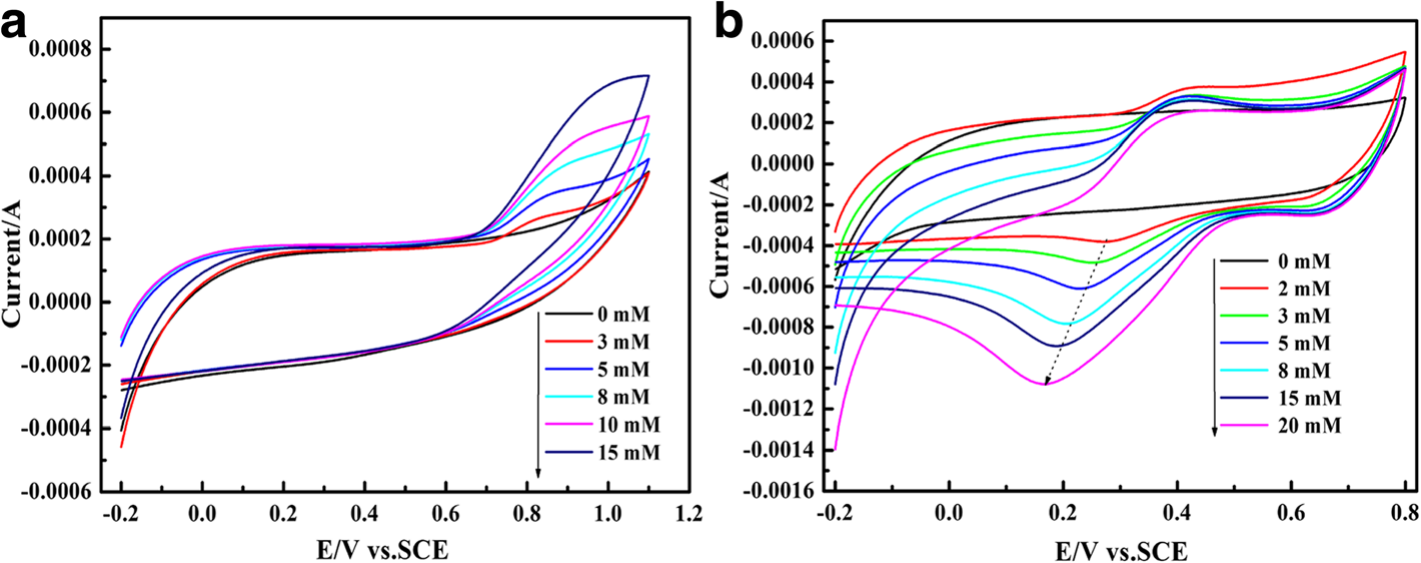
Cyclic voltammograms of PEDOT/Au/GO/GCE in 0.025 M PBS (pH = 6.86) solution containing nitrite (**a**) and 0.1 M H_2_SO_4_ solution containing iodate (**b**)

Figure 7 shows cyclic voltammograms of PEDOT/Au/GO/GCE in 0.025 M PBS (pH = 6.86) solution containing nitrite (Fig. 7a and 0.1 M H_2_SO_4_ solution containing iodate (Fig. 7b), respectively. The peak current increases with the increase of nitrite concentration (3 to 15 mM) and iodate concentration (2 to 20 mM), respectively. As seen in Fig. 7a, there is broad oxidation peak at about 0.82 V, which can be assigned to the conversion of NO_2_^−^ to NO_3_^−^ through a two-electron oxidation process [49]. In the case of the iodate (Fig. 7b), the reduction peak currents increases and the peak potential slightly shifts from 300 to 160 mV, which can be attributed to the rapid reduction of IO_3_^−^ to I^−^ [48].

**Fig. 8:**
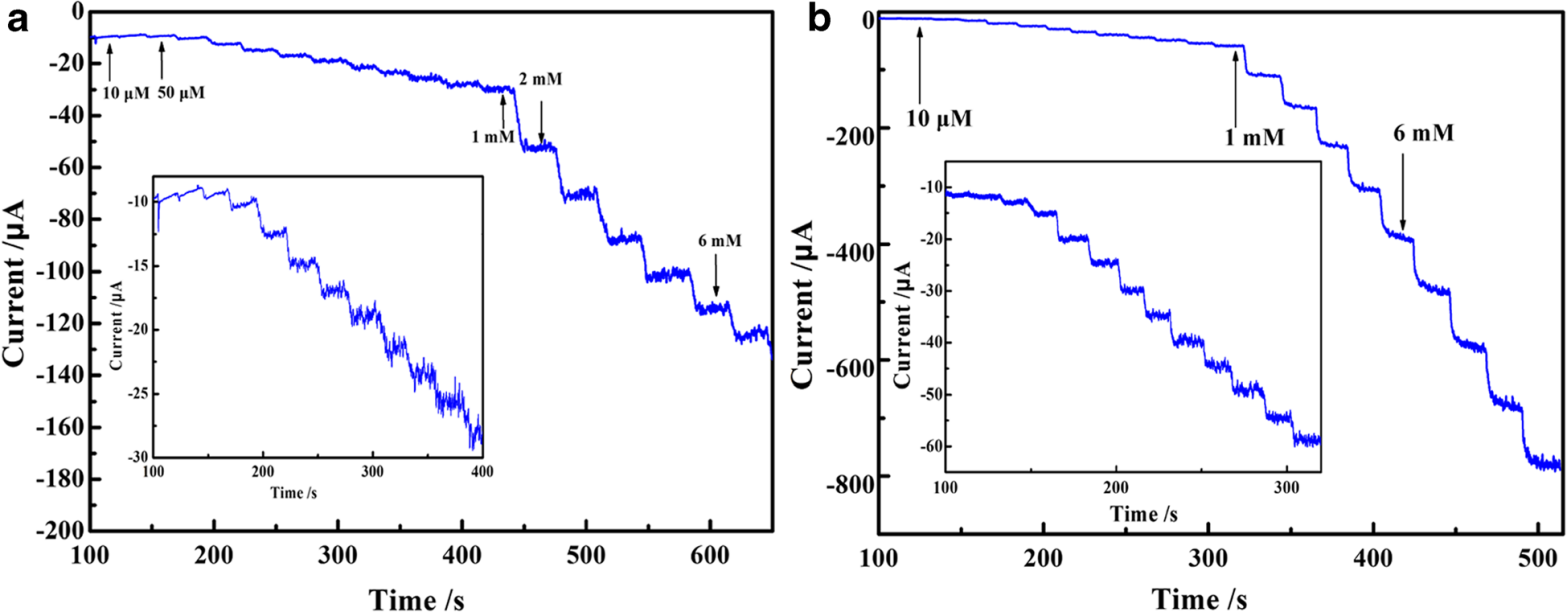
Steady-state catalytic current-time response of PEDOT/Au/GO/GCE with successive addition of 1.0 × 10^−5^, 1.0 × 10^−4^, and 1.0 × 10^−3^ M nitrite (**a**) and iodate (**b**)

Figure 8 shows the steady-state catalytic current-time response of PEDOT/Au/GO/GCE with successive addition of 1.0 × 10^−5^, 1.0 × 10^−4^, and 1.0 × 10^−3^ M nitrite (Fig. 8, potential controlled at 0.78 V) and iodate (Fig. 8b, potential controlled at − 0.25 V), respectively. As shown in Fig. 8, a well-defined response is observed under the successive addition of 1.0 × 10^−5^, 1.0 × 10^−4^, and 1.0 × 10^−3^ M nitrite and iodate, respectively.

**Fig. 9:**
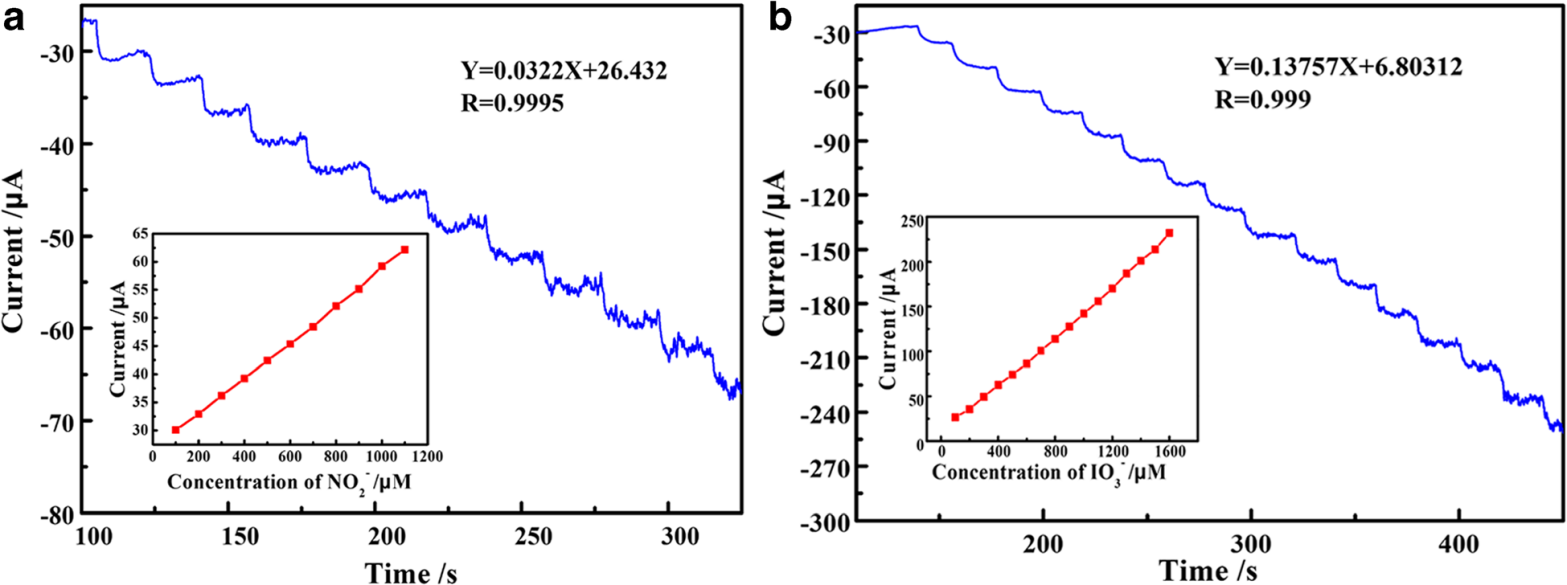
Steady-state catalytic current-time response of PEDOT/Au/GO/GCE with successive addition of 1.0 × 10^−3^ M nitrite (**a**) and iodate (**b**)

Figure 9 shows the steady-state catalytic current-time response of PEDOT/Au/GO/GCE with successive addition of 1.0 × 10^−3^ M nitrite (Fig. 9a, potential controlled at 0.78 V) and iodate (Fig. 9b, potential controlled at − 0.25 V). The results from Fig. 9 show that the detection of both nitrite and iodate has better steady-state catalytic current in the range of 100–1000 μM, and the response time is about 4 s after each addition of nitrite and iodate, respectively. The plots of chronoamperometric currents vs. ion concentration (insets in Fig. 9) further indicate a good linear relationship exists between peak current and the concentration in the range of 100–1000 μM with the linear equations of *I*_(μA)_ = 0.0322 C + 26.422 (*R* ^2^ = 0.9995) and *I*_(μA)_ = 0.13757C + 6.80312 (*R* ^2^ = 0.999) for nitrite and iodate, respectively. Most importantly, the detection of nitrite and iodate by PEDOT/Au/GO/GCE exhibits a step response and has an ideal current response for electrochemical detection for nitrite and iodate with loading of small amount of composite (5 μL from 30 mg/L) on glassy carbon electrode. Besides, the low detection limit are estimated to be 0.53 μM and 0.62 μM (S/N = 3) for nitrite and iodate, respectively.

The comparisons for the parameters of nitrite and iodate detection by various chemically modified electrodes are listed in Table 1. The comparison results show that the response of PEDOT/Au/GO/GCE modified electrode has a lower current (9.59 μA) than that (17.5 μA) of MWNT-PAMAM-Chit in addition of 10 μM nitrite. However, the current response of PEDOT/Au/GO/GCE for addition of 10 μM nitrite is higher than that (0.3 μA) of Nano-Au/P3MT/GCE. In addition, the current response of PEDOT/Au/GO composite is 11.47 μA for addition of 10 μM iodate, which also gives a better proof that the PEDOT/Au/GO/GCE-modified electrode is suitable [25] for the detection for iodate.

**Table 1:** Comparison of linear range and response current of nitrite and iodate for different sensors

Electrode	Linear range (μM)		Response current (μA)^a^		Reference
	Nitrite	Iodate	Nitrite	Iodate	
Pt-Fe(III)/GCE	43.5–971	_	0.79	_	[12]
PQ/MWNTs	_	1–2000	_	0.32	[15]
Nano-Au/P3MT/GCE	10–1000	_	~ 0.3	_	[21]
PANI/MWNTs/gold	5–15,000	_	2.8	_	[34]
SWNTs/ssDNA/GCE	10–300	_	2.3	_	[48]
PEDOT/Au/GNP	10–10,000	10–10,000	9.59	11.47	This work

^a^At the detection concentration 10 μM

**Fig.10:**
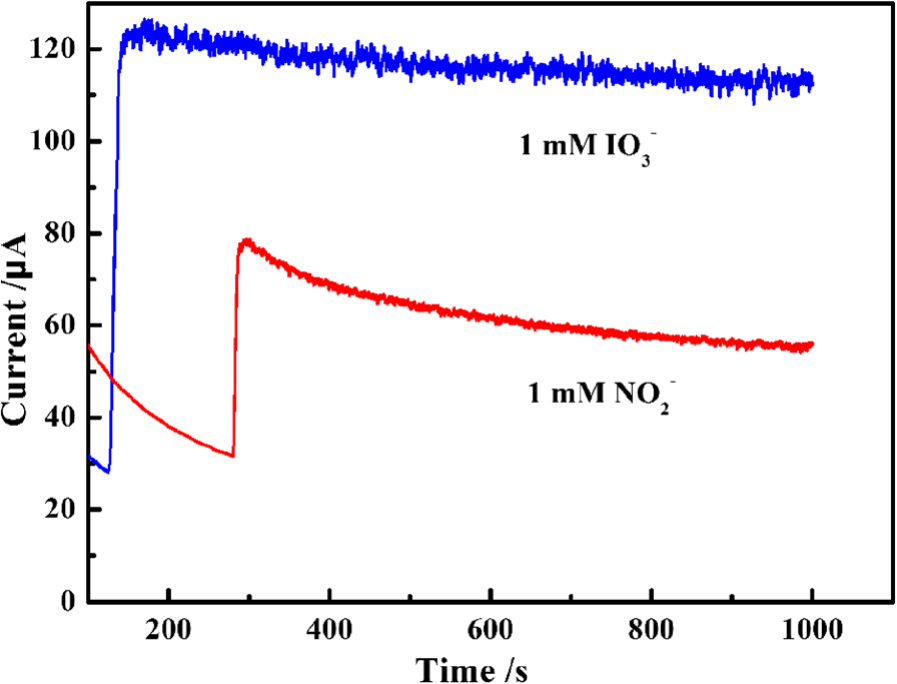
A recorded amperometric of PEDOT/Au/GO/GCE in 1 mM of nitrite (**a**) and iodate (**b**) during long period time 1000 s

Figure 10 shows the PEDOT/Au/GO/GCE composite modified electrode imparts higher stability onto amperometric measurements of analyte (1.0 mM nitrite or 1.0 mM iodate) during prolonged 1000 s experiment. The response remains stable throughout the experiment, indicating no inhibition effect of iodate and its reduction products for modified electrode surface. However, comparing with iodate, the response remains unstable in the case of nitrite.

**Fig. 11:**
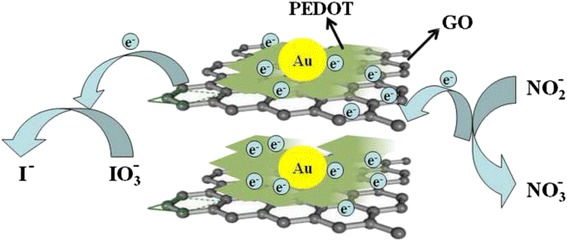
Mechanisms of the direct electron transfer between ion (nitrite or iodate) and GCE through the PEDOT/Au/GO composite

Figure 11 shows the mechanisms of the direct electron transfer between ion (nitrite or iodate) and GCE (glassy carbon electrode) through the PEDOT/Au/GO/GCE composite. As depicted in Fig. 11, the shale-like PEDOT can incorporate with GO to form a lamellar structure, which can lead a large surface area for uniform distribution of Au nanoparticles. Furthermore, the generated electrons will conduct to GCE via the shortest resistance path through highly conductive GO dispersed in composite as illustrated in Fig. 11. However, without GO, the electrons will have to go through PEDOT medium, which has considerable resistance that causes significant potential drop and much lower electron transfer rate. Therefore, GO plays an important role in facilitating the electron exchange between ion (nitrite or iodate) and GCE because it forms conductive matrix leading to reduced electrical resistance paths.

### Real Sample Analysis

In order to validate/test the practical application of the modified electrode, the PEDOT/Au/GO/GCE was applied for detection of nitrite concentration in tap water with standard addition method. A certain volume of samples was added to electrochemical cell for the determination of nitrite by amperometric determination. As shown in Table 2, the recovery of the sample ranged from 98.4 to 104.3%. Therefore, the PEDOT/Au/GO/GCE could be used for the detection of nitrite in water sample.

**Table 2:** Determination of nitrite tap water

Sample	Added (μM)	Found (μM)	Recovery (%)
1	30	30.8	102.6
2	60	62.6	104.3
3	100	98.4	98.4

## Conclusion

A ternary composite of PEDOT/Au/GO for promising electrochemical sensor was synthesized by solid-state heating method. The results revealed that the shale-like morphology of PEDOT might bring some possibility for formation of lamellar structures from incorporation of PEDOT in GO matrix, which could lead a large surface area for uniform distribution of Au nanoparticles. Therefore, the synergistic effect between PEDOT, GO, and Au nanoparticles as well as the large contact surface area of composite led the PEDOT/Au/GO composite display a strong electrocatalytic activity toward the oxidation of nitrite and reduction of iodate. And the current responses of the detection of nitrite and iodate were high enough to achieve an obvious step response. Furthermore, the PEDOT/Au/GO composite had an ideal current response for electrochemical detection for nitrite and iodate with loading of small amount of composite (5 μL from 30 mg/L) on glassy carbon electrode.
